# ﻿Four new species underline the hidden diversity and long-range dispersal in *Deltoxenos* Benda, Pohl, Nakase, Beutel & Straka (Strepsiptera, Xenidae)

**DOI:** 10.3897/zookeys.1254.160903

**Published:** 2025-09-30

**Authors:** Daniel Benda, Hans Pohl, Rolf Beutel, Jakub Straka

**Affiliations:** 1 Department of Entomology, National Museum of the Czech Republic, Prague, Czech Republic Charles University Prague Czech Republic; 2 Department of Zoology, Faculty of Science, Charles University, Prague, Czech Republic National Museum of the Czech Republic Prague Czech Republic; 3 Institut für Zoologie und Evolutionsforschung, Friedrich-Schiller-Universität, Jena, Germany Friedrich-Schiller-Universität Jena Germany

**Keywords:** Cephalothorax, *
Delta
*, morphology, parasite association, potter wasps, taxonomy, wasp parasites

## Abstract

Four new species of Strepsiptera from the genus *Deltoxenos* Benda, Pohl, Nakase, Beutel & Straka (Xenidae) are described based on the female cephalothoraces. These include the first *Deltoxenos* species from the Indomalayan and Australasian regions. New host species and genera for Xenidae – *Phimenes solomonis* (Vecht, 1959) and *Pareumenes quadrispinosus* (Saussure, 1855) – are recorded as strepsipteran hosts for the first time. Diagnoses and descriptions of female cephalothoraces are presented for all four newly described species of *Deltoxenos*, with brief diagnoses for *Deltoxenos bidentatus* Pasteels, 1950 and *Deltoxenos rueppelli* Kinzelbach, 1971. The known distribution of *D. bidentatus* and *D. rueppelli* are significantly expanded, and a notably broader host range documented for the latter. Diagnostic characters and geographic distribution are discussed in the context of a recent long-range dispersal event from Afrotropics to the Australasian region.

## ﻿Introduction

Xenidae are wasp endoparasites specialised on four families, Crabronidae, Bembicidae, Sphecidae, and Vespidae (Benda et al. 2021). The family originated relatively late, approximately 50–60 million years ago (McMahon et al. 2011). Within the Strepsiptera, they belong to Stylopidia, a clade containing more than 97% of species of the order and parasitising only neopteran pterygote insects (Pohl and Beutel 2008). Benda et al. (2022b) provided a generic revision and a detailed checklist of Xenidae, and delimited 13 genera based on previous molecular phylogenetic studies (Benda et al. 2019, 2021). Xenidae and its sister taxon Stylopidae are the groups with the highest degree of specialisation and specificity in Strepsiptera (Pohl and Beutel 2008; Nakase and Kato 2013; Jůzová et al. 2015). Xenidae are mainly characterised by unique characters of the first instar larva, which is the host-seeking life stage. These features enhance the attachment capacity to the smooth body surface of the wasp hosts. This includes enlarged and round tarsal adhesive pads and filamentous cuticular outgrowths of the labium, which strongly increase its wettability (Pohl and Beutel 2004, 2008).

*Deltoxenos* Benda, Pohl, Nakase, Beutel & Straka is a recently described genus with eight species (Benda et al. 2022b, 2024). It is a lineage of Afrotropical origin, widely distributed in the Old World and it extends into the Australasian region, although it is not found directly in Australia (Benda et al. 2019). Previously, these species were classified in the genus *Pseudoxenos* Saunders based on the hypothesis that all parasites of solitary wasps belong to this genus (Kinzelbach 1971b). However, based on molecular phylogenetic studies, it was found that the genus *Pseudoxenos*, parasitising solitary bees, split into four lineages, which are now considered separate genera (Benda et al. 2019, 2022b). This lineage of *Deltoxenos* utilises a diverse range of hosts from in Odynerini and Eumenini (Vespidae: Eumeninae). Interestingly, the parasitism of Odynerini occurred deep in the evolutionary history of xenids and they are used as hosts by several genera, whereas parasitism of Eumenini evolved relatively late and is restricted to the genus *Deltoxenos* (Benda et al. 2019, 2022b). The lineage that parasitises Eumenini is a subordinate group within the genus *Deltoxenos*. The switch from Odynerini to Eumenini occurred in the Afrotropics and was associated with the expansion to the Palearctic, Indomalayan, and Australasian regions in the recent history of the group (6 Mya, 3–11 Mya) (Benda et al. 2019).

To date, only two species of *Deltoxenos* parasitising Eumenini have been described. The first species, *Deltoxenos bidentatus* (Pasteels), was described from *Afreumenes melanosoma melanosoma* (Saussure) (as *Eumenes melanosomus decipiens* Kirby) in Central Africa (Pasteels 1950). In the last-mentioned study, the species was classified in the genus *Pseudoxenos* as a parasite of solitary wasps. Later, *Deltoxenos rueppelli* (Kinzelbach) was described from *Delta fenestrale* (Saussure) (as *Eumenes fenestralis* Saussure) in East Africa (Eritrea) based on the female cephalothorax and the first instar larva (Kinzelbach 1971a). In a comprehensive study on African species of Strepsiptera, Luna de Carvalho (1978) provided additional information on the distribution and other hosts of both species.

Although *D. bidentatus* and *D. rueppelli* were described relatively late, several stylopised representatives of Eumenini are listed in the earlier literature containing checklists of parasitised hosts (Salt 1927; Salt and Bequaert 1929; Hofeneder and Fulmek 1942). These records were largely adopted by Luna de Carvalho (1978) in his study of African strepsipterans, where he attributed the known distribution and host associations to both described species.

Although *Deltoxenos* as a recently described genus of African origin, currently includes only two species parasitising Eumenini in Africa, it is probably the genus with the highest number of undescribed species within the family Xenidae (Benda et al. 2022b). In the present study, we contribute to narrowing this taxonomic gap by describing four new species of *Deltoxenos* from three biogeographic regions. We provide diagnoses and descriptions of the species in accordance with a previous phylogenetic study (Benda et al. (2021) and a revised generic classification based on the morphology of the female cephalothorax (Benda et al. 2022b).

## ﻿Material and methods

The material of Strepsiptera from four host genera – *Delta* Saussure, *Afreumenes* Bequaert, *Phimenes* Giordani Soika, and *Pareumenes* Saussure comprised a total of 46 females, 6 empty male puparia and 3 male puparia with attached cephalotheca. Material from the following public and private collections was examined:

**JSPC** Jakub Straka personal collection, Prague, Czech Republic;

**KUNHM** Natural History Museum, Division of Entomology, University of Kansas, Lawrence, Kansas, USA;

**NMPC**National Museum of the Czech Republic, Prague, Czech Republic;

**OLML**Oberösterreichisches Landesmuseum, Linz, Austria.

A total of 39 females of *Deltoxenos* were used for dissection, the others were observed in the host. The host individuals were relaxed in water vapour and then immediately dissected. The dissected endoparasitic females and male puparia were removed from the host’s body and cleared using a mixture of lysis buffer ATL and proteinase K (Qiagen) at 56 °C for several hours. The cleared specimen was cleaned in distilled water several times and then stored in a vial with 96% ethanol. The female cephalothoraces were air-dried or dried with absolute ethanol and hexamethyldisilazane (HMDS method) (Heraty 1998) to prevent the cuticle from collapsing during the drying process. The female bodies were extracted from the cephalothoraces before drying. After this step, the dried specimens were glued onto card mounting points, which were then pinned.

The width and length of the female cephalothorax, the female head capsule, and the male cephalotheca were measured using a Leica S9D Stereomicroscope with a calibrated ocular micrometre. The cephalothorax length was measured from the apex of the clypeal lobe to the constriction of abdominal segment I; the cephalothorax width is the maximum distance between its lateral margins.

The general habitus of stylopised host specimens and the host abdomen with protruding strepsipterans were documented with multifocus images, taken with Canon EOS 550D or 70D cameras equipped with EF 50mm and MP-E 65 mm macro lenses. Lateral lights and a diffuser were used.

For the documentation of the original colouration of the female larval cephalothorax and the male cephalotheca, specimens glued to card mounting points were used. They were photographed with a Canon EOS 7D digital SLR equipped with a Canon MP-E 65mm macro lens (Canon, Krefeld, Germany) fitted with a StackShot macro rail (Cognisys, Traverse City, MI, USA). Each specimen was illuminated with two flashlights (Yongnuo Photographic Equipment, Shenzhen, China) fitted to a transparent cylinder for even and soft light. Zerene Stacker (Zerene Systems LLC, Richland, USA) was used to process stacks of images with different focus.

The dried cephalothoraces glued to a card point were mounted on a specimen holder by adhesive carbon tabs. The specimens were not sputter coated with gold. SEM images were taken using a Hitachi S-3700N environmental electron microscope (Hitachi, Tokio, Japan) at the Department of Palaeontology, National Museum of the Czech Republic in Prague.

All images were processed and arranged into plates with Adobe Photoshop® CS5 (Adobe System Incorporated, San Jose, USA) software. CorelDraw® X8 (CorelDraw Corporation, Ottawa, ON, Canada) was used for the lettering of the plates.

The terminology used for the female cephalothorax is adopted from Benda et al. (2024), Benda et al. (2022b), Richter et al. (2017), Löwe et al. (2016), and Kinzelbach (1971b). New appropriate terminology was developed for morphological characters without specific designations. The cephalothorax is described in morphological orientation in figures although their functional orientation in the host’s body is inverted. Abbreviations: ♀ – female, **MP** – male puparium, **EMP** – empty male puparium. In the sections Material examined, the samples are sorted based on the country where they were collected.

## ﻿Results

### ﻿ Deltoxenos bidentatus

Taxon classificationAnimaliaStrepsipteraXenidae

Pasteels, 1950

B3533DDB-CD57-581A-86CA-2CDE0290E3C6

#### Material examined.

• ♀ (NMPC), Central African Republic: Mbaiki 55km NW, 510m, 23.xii.2008, host: *Afreumenes* sp., J. Halada lgt., voucher code: PsAf1 • ♀ (NMPC), Central African Republic: Mbaiki 150km NW, 14.vi.2009, host: Afreumenes cf. aethiopicus (Saussure, 1852), J. Halada lgt., voucher code: PsDe3 • 4♀ (KUNHM), Malawi: Mlanje env., 06.iv.1967, host: *Afreumenes aethiopicus* (Saussure, 1852), Ch. Michener lgt., voucher code: PsAe1.

#### Diagnosis of female cephalothorax.

Size of cephalothorax: length 1.24–1.48 mm, width 1.16–1.48 mm. Cephalothorax triangular, meso- and metathorax not elongated; apical maxillary region projecting beyond mandibular apex (Benda et al. 2022b: fig. 47C). Mandibles relatively small compared to head capsule (Benda et al. 2022b: fig. 49A).

#### Hosts.

*Afreumenes melanosoma* (Saussure 1852) (*Eumenes melanosomus decipiens* Kirby), type host; *Afreumenes aethiopicus* (Saussure, 1852) (Pasteels 1950; Luna de Carvalho 1978); Afreumenes cf. aethiopicus (Saussure, 1852) (Benda et al. 2022b); *Afreumenes* sp. (this study).

#### DNA barcode sequences (GenBank).

MN914583.1 (voucher code: PsAf1), MK431201.1 (voucher code: PsDe3) (Benda et al. 2021).

#### Phylogenetic relationships.

The earliest diverging species in lineage of *Deltoxenos* parasitising Eumenini (Vespidae) (Benda et al. 2021).

#### Distribution.

Democratic Republic of Congo (type locality); Liberia (Pasteels 1950, Luna de Carvalho 1978); Central African Republic (Benda et al. 2021); Malawi (Benda et al. 2022b).

#### Comments.

Benda et al. (2021) lists *Delta tropicale* (Saussure, 1852) as the host of this species, but it was identified as *Afreumenes* sp.

### ﻿ Deltoxenos hajeki

Taxon classificationAnimaliaStrepsipteraXenidae

Benda & Straka sp. nov.

A73E9ECF-3F46-5220-96AD-DC5C8A313E53

https://zoobank.org/9FB5BC0B-9F32-4BD2-A52D-7FC7B644F323

**Figure 1.**
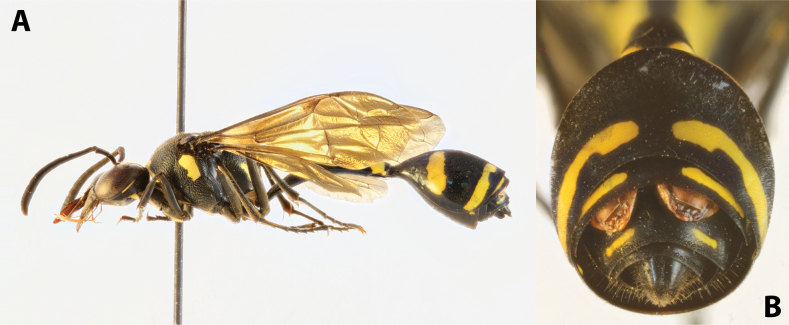
*Deltoxenos hajeki* Benda & Straka, sp. nov., host, female cephalothorax. A. Female of *Phimenes solomonis* (Vecht, 1959), stylopised by *D. hajeki* sp. nov., lateral view; B. Detail of host abdomen of *P. solomonis*, with two female cephalothoraces inside.

**Figure 2.**
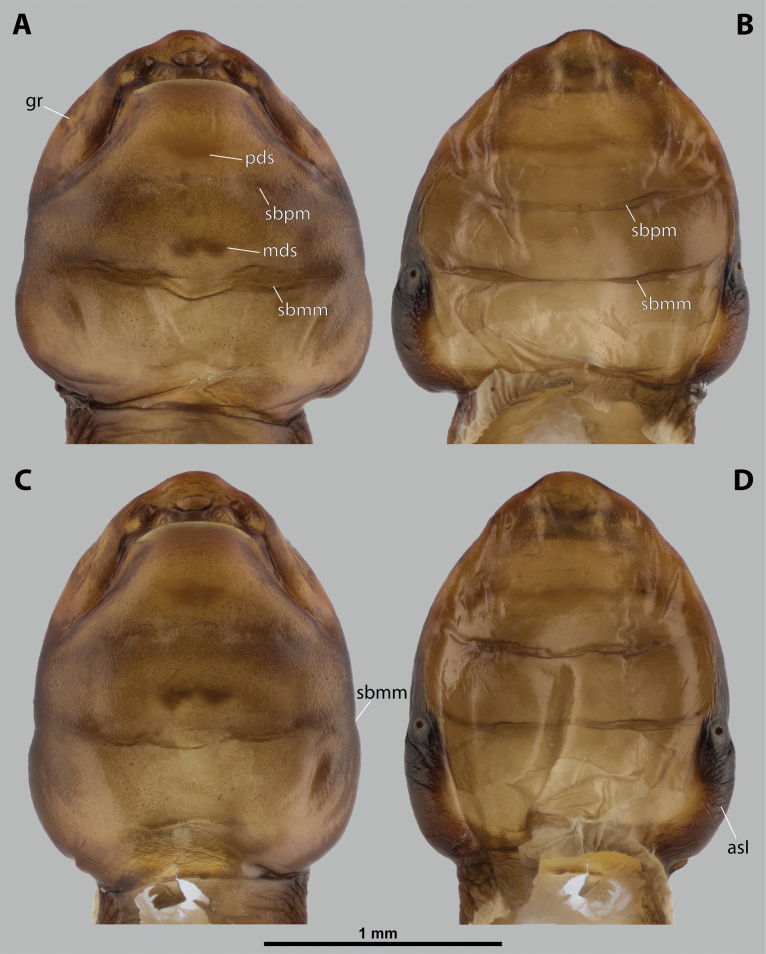
*Deltoxenos hajeki* Benda & Straka, sp. nov., female, cephalothorax, photomicrographs. A, B. Paratype, ventral and dorsal sides of cephalothorax; C, D. Holotype, ventral and dorsal sided of cephalothorax. Abbreviations: asI – abdominal segment I, gr – longitudinal grooves, mds – mesosternal dark spot, pds – prosternal dark spot, sbmm – segmental border between mesothorax and metathorax, sbpm – segmental border between prothorax and mesothorax.

**Figure 3.**
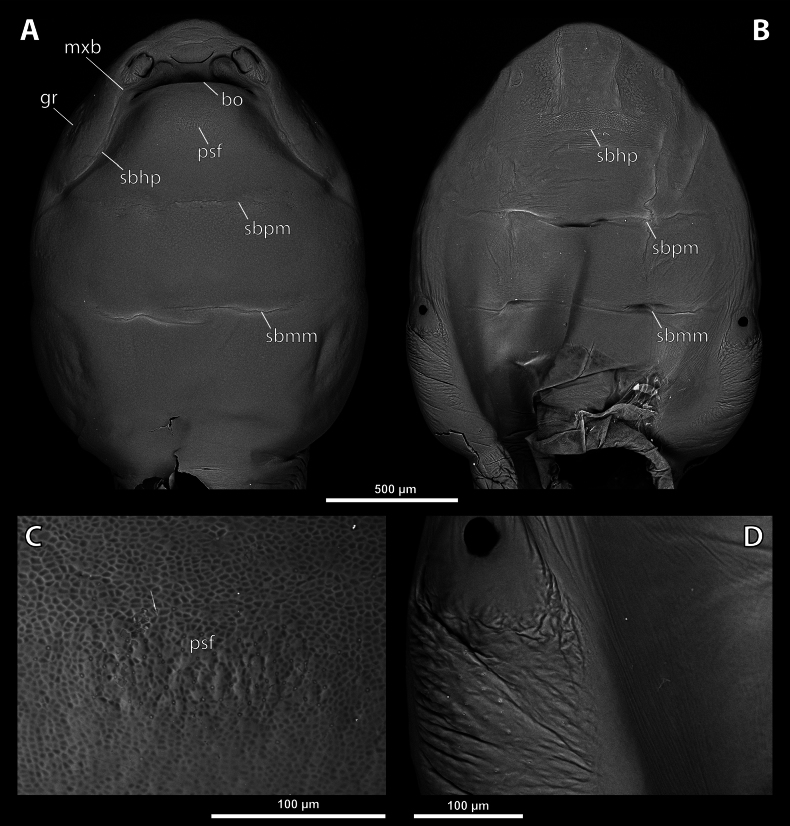
*Deltoxenos hajeki* Benda & Straka, sp. nov., holotype, female cephalothorax, SEM micrographs. A. Ventral side; B. Dorsal side; C. Detail of prosternum; D. Posterolateral part of cephalothorax below spiracle, dorsal side. Abbreviations: bo – birth opening, gr – longitudinal grooves, mxb – maxillary base, psf – prosternal sensillary field, sbhp – segmental border between head and prothorax, sbmm – segmental border between mesothorax and metathorax, sbpm – segmental border between prothorax and mesothorax.

**Figure 4.**
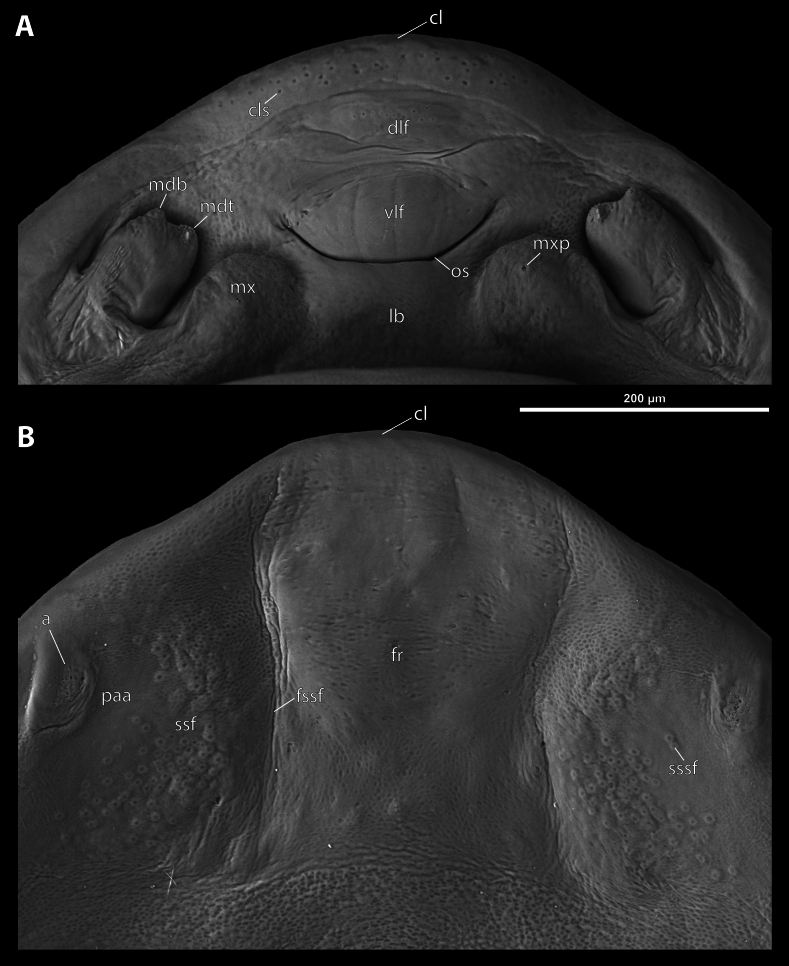
*Deltoxenos hajeki* Benda & Straka, sp. nov., holotype, female cephalothorax, SEM micrographs. A. Detail of head capsule, ventral side; B. Detail of head capsule, dorsal side. Abbreviations: a – vestigial of antenna, cl – clypeus, cls – clypeal sensillum, dlf – dorsal labral field of labral area, fr – frontal region, fssf – furrow of supra-antennal sensillary field, lb – labial area, mdb – mandibular bulge, mdt – mandibular tooth, mx – vestige of maxilla, mxp – vestige of maxillary palp, os – mouth opening, paa – periantennal area, ssf – supra-antennal sensillary field, sssf – sensillum of supra-antennal sensillary field, vlf – ventral labral field of labral area.

Figs 1A, B, 2A–D, 3A–D, 4A, B

#### Type material.

***Holotype*** • ♀ (NMPC), Solomon Islands: Guadalcanal, 4.5 km S of Barana, 275m, 6.xii.2013, host: *Phimenes solomonis* (Vecht, 1959), J. Hájek lgt. ***Paratype*** • ♀ (NMPC), data the same as holotype, from the same host specimen as holotype (Fig. 1).

#### Diagnosis of female cephalothorax.

This species is easily distinguished from other representatives of the genus *Deltoxenos* by a conspicuous dark spot posteriorly on the prosternum (pds, Fig. 2A) and a double dark spot (mds, Fig. 2A) situated mesally on the mesosternum. The cuticular surface of the prosternal and mesosternal dark spots is smooth, without any papillae or reticulation. It differs from *D. indonesiensis* and *D. reginus* by well-developed and prominent maxillae, separated from the labial area (mx, Fig. 4A), and by the mostly pale colouration of the prosternum and mesosternum. It differs from *D. rueppelli* by a rounded and protruding clypeal lobe (cl, Fig. 12). From *D. maceki* it can be distinguished by ventrally exposed clypeal sensilla (cls, Fig. 4A) which are mainly concentrated anteriorly on clypeal lobe in *D. maceki*. The antennal torulus is slightly reduced but still present (Fig. 4B) while it is completely absent in *D. maceki* (Fig. 11C).

#### Description of female cephalothorax.

***Shape and colouration*.** Size of holotype cephalothorax: length 1.74 mm, width 1.42 mm (Fig. 2C). Size of paratype cephalothorax: length 1.62 mm, width 1.52 mm (Fig. 2A). Cephalothorax variable in size, but always distinctly longer than wide; paratype shorter and wider than holotype. Constriction at pro-mesothoracic segmental border not visible; meso-metathoracic segmental border conspicuously constricted laterally (sbmm, Fig. 2C). Abdominal segment I not protruding laterally, corner below spiracles rounded. Anterior head margin rounded, slightly protruding from remaining head capsule. Thorax elongated, very slightly widening posteriorly. Cephalothorax with conspicuously contrasting pale and dark colour pattern but predominantly pale.

***Head capsule*.** Length proportion of head/cephalothorax 0.44–0.45 including lateral cephalic extension. Colouration forming specific pattern with predominantly pale parts and dark brown mandible and labium. Surface of lateral extensions at site of reduced compound eyes smooth, laterally with conspicuous longitudinal grooves visible on SEM images (gr, Fig. 3A), with dark colouration (gr, Fig. 2A). Clypeal area well delimited from labral area, arcuate, clypeal lobe slightly protruding from head capsule. Surface completely smooth with slightly > 44 distinctly exposed sensilla mainly concentrated on clypeal lobe on ventral side (cls, Fig. 4A). Dorsal side of clypeal area smooth and lacking sensilla (cl, Fig. 4B). Border between clypeal and frontal region indistinct but still present. Frontal region smooth, slightly reticulated (fr, Fig. 4B). Segmental border between head and prothorax indicated by indistinct mesal furrow on dorsal side (sbhp, Fig. 3B) and by dorsal transverse stripe of reticulated cuticular surface on frontal region. Head and prothorax distinctly separated by birth opening ventromedially (bo, Fig. 3A) and laterally by suture (sbhp, Fig. 3A).

***Supra*-*antennal sensillary field*.** Reticulated to completely smooth, with dispersed sensilla inserted in cavities (sssf, Fig. 4B). Distinctly delimited by furrow on medial side (fssf, Fig. 4B). Surface of supra-antennal sensillary field and frontal region with different sculpture.

***Antenna*.** Preserved as well-defined area, with numerous vestigial sensilla, distinct rounded plates and inconspicuous cavities (a, Fig. 4B). Antennal torulus reduced but still present as furrow between antenna and periantennal area. Periantennal area slightly expanded, smooth (paa, Fig. 4B).

***Labrum*.** Ventral field wider than long, elliptic. Dorsal field very slightly arcuate, flat, not raised, laterally not narrower than medially, 4× wider than long in midline (vlf, dlf, Fig. 4A). In holotype, dorsal field with 11 sensilla inserted in cavities (dlf, Fig. 4A).

***Mandible*.** Mandibles anteromedially directed at angle of 40–45° (45° in holotype), enclosed within mandibular capsule. Mandibular bulge distinctly raised, elongated, anteriorly directed, with several sensilla (mdb, Fig. 4A). Cuticle of mandible smooth medially, laterally with longitudinal furrows. Mandibular tooth not curved, pointing dorsally, armed with many spines (mdt, Fig. 4A).

***Maxilla*.** Well-developed and prominent, separated from labial area (mx, Fig. 4A). Mostly with pale colouration, cuticle smooth or slightly reticulated. Apical maxillary region not projecting beyond mandibular apex. Basal part connected with labium and not overlapping with mandible (mxb, Fig. 3A). Vestige of palp present, located medially on ventral side of maxilla (mxp, Fig. 4A). Maxillary base distinctly produced anterolaterally as submaxillary groove. Space between prothoracic extension and head extended (sbhp, mxb, Fig. 3A).

***Labium*.** Labial area between maxillae distinct, delimited anteriorly by mouth opening and posteriorly by birth opening (lb, Fig. 4A). Flat, approximately as long as wide. Cuticular surface smooth to very slightly reticulated.

***Mouth opening*.** Widely arcuate but not semicircular, sclerotised along margin (os, Fig. 4A).

***Thorax*.** Pro-mesothoracic and meso-metathoracic borders visible ventrally as slightly imprinted mesal furrows (sbpm, sbmm, Figs 2A, 3A). On dorsal side separated by conspicuous dark mesal furrows, distinctly contrasting with pale thoracic segments (sbpm, sbmm, Figs 2B, 3B). Border between metathorax and abdomen indicated by ventral ridge on ventral side or indicated by change in colour and cuticular sculpture. Cuticle of thoracic segments on ventral side mostly reticulated, uniformly scattered with inconspicuous or more distinct pigmented papillae, except dark spots. Prosternum differentiated, anteriorly with field of dozens of sensilla (psf, Fig. 3A, C), posteriorly with conspicuous dark spot (pds, Fig. 2A). Mesosternum almost completely covered with dark papillae except mesal double dark spot (mds, Fig. 2A). Cuticular surface of prosternal and mesosternal dark spots smooth, without any papillae or reticulation. All thoracic segments dorsally pale, but darker laterally. Meso- and metathorax transverse, rarely slightly elongated.

***Abdominal segment I and spiracles.*** Setae and cuticular spines present on lateral region of abdominal segment I posterior to spiracle (Fig. 3D). Spiracles on posterior ~2/5 of cephalothorax, very slightly elevated, with lateral or dorsolateral orientation. Cephalothoracic part of abdominal segment I below spiracles dark brown on both sides (asI, Fig. 2D).

#### Host.

*Phimenes solomonis* (Vecht, 1959)

#### Phylogenetic relationships.

Unknown.

#### Distribution.

Solomon Islands.

#### Etymology.

Named after Jiří Hájek (National Museum of the Czech Republic, Prague), a dear colleague and expert on aquatic beetles, who collected the type material.

### ﻿ Deltoxenos indonesiensis

Taxon classificationAnimaliaStrepsipteraXenidae

Benda & Straka sp. nov.

4D87D5F9-26B9-5A2A-88E7-D1FD4C55C617

https://zoobank.org/42756AEF-D114-4EED-9C98-FA3681601C94

**Figure 5.**
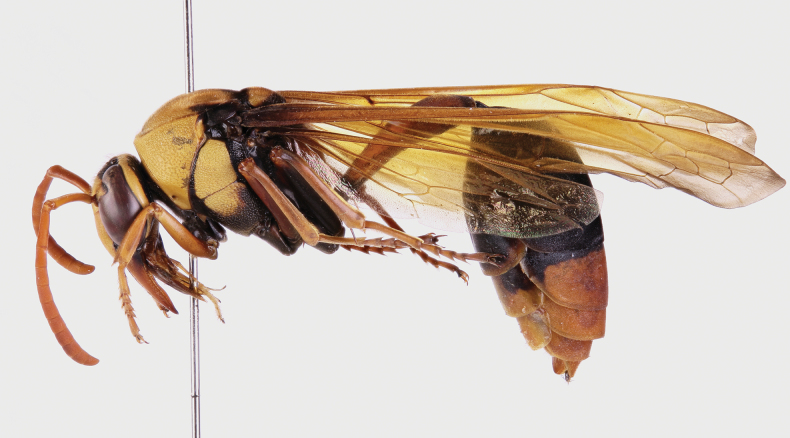
Female of *Delta pyriforme miraculum* Gusenleitner, 2008, host of *Deltoxenos indonesiensis* Benda & Straka, sp. nov., photographed after removal of the female cephalothorax.

**Figure 6.**
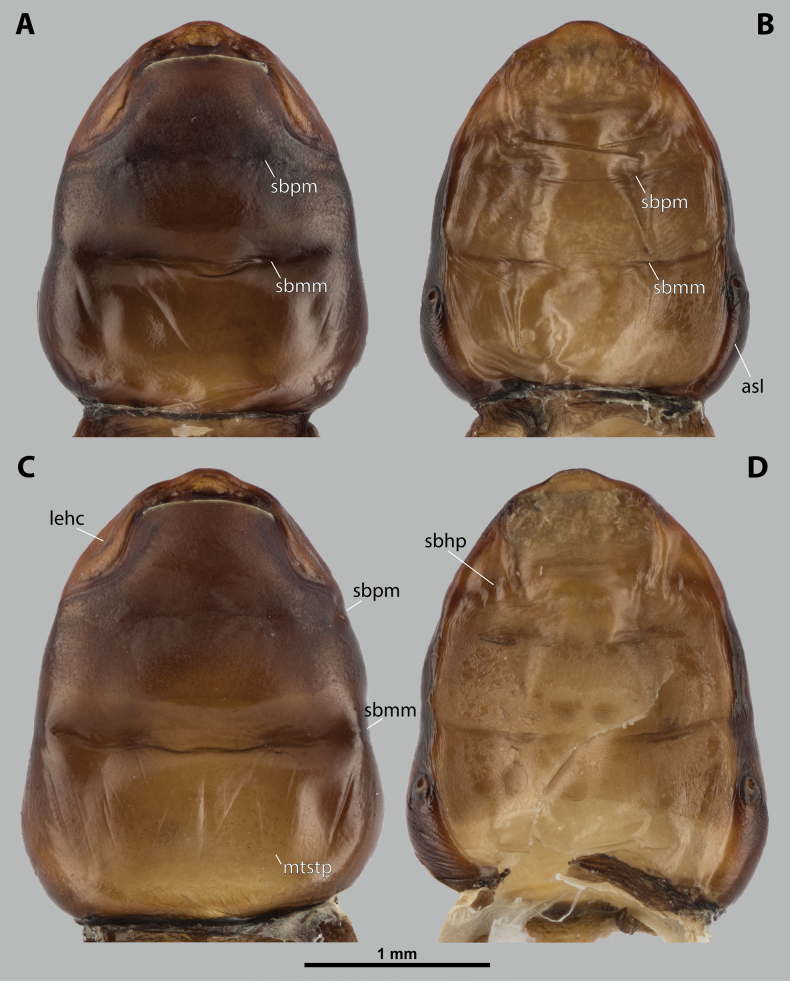
*Deltoxenos indonesiensis* Benda & Straka, sp. nov., female, cephalothorax, photomicrographs. A, B. Paratype, voucher code: PsMr3b, ventral and dorsal side of cephalothorax; C, D. Paratype, voucher code: PsMr2, ventral and dorsal side of cephalothorax. Abbreviations: asI – abdominal segment I, lehc – lateral extension of head capsule, mtstp – metasternal papilla, sbhp – segmental border between head and prothorax, sbmm – segmental border between mesothorax and metathorax, sbpm – segmental border between prothorax and mesothorax.

**Figure 7.**
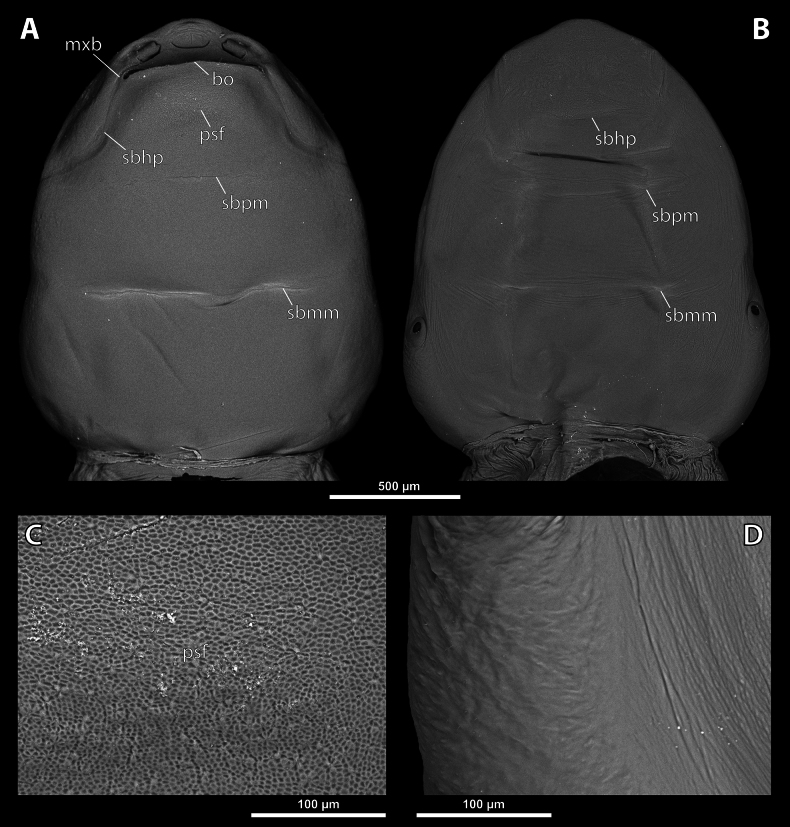
*Deltoxenos indonesiensis* Benda & Straka, sp. nov., paratype, voucher code: PsMr3b, female cephalothorax, SEM micrographs. A. Ventral side; B. Dorsal side; C. Detail of prosternum; D. Posterolateral part of cephalothorax below spiracle, dorsal side. Abbreviations: bo – birth opening, mxb – maxillary base, psf – prosternal sensillary field, sbhp – segmental border between head and prothorax, sbmm – segmental border between mesothorax and metathorax, sbpm – segmental border between prothorax and mesothorax.

**Figure 8.**
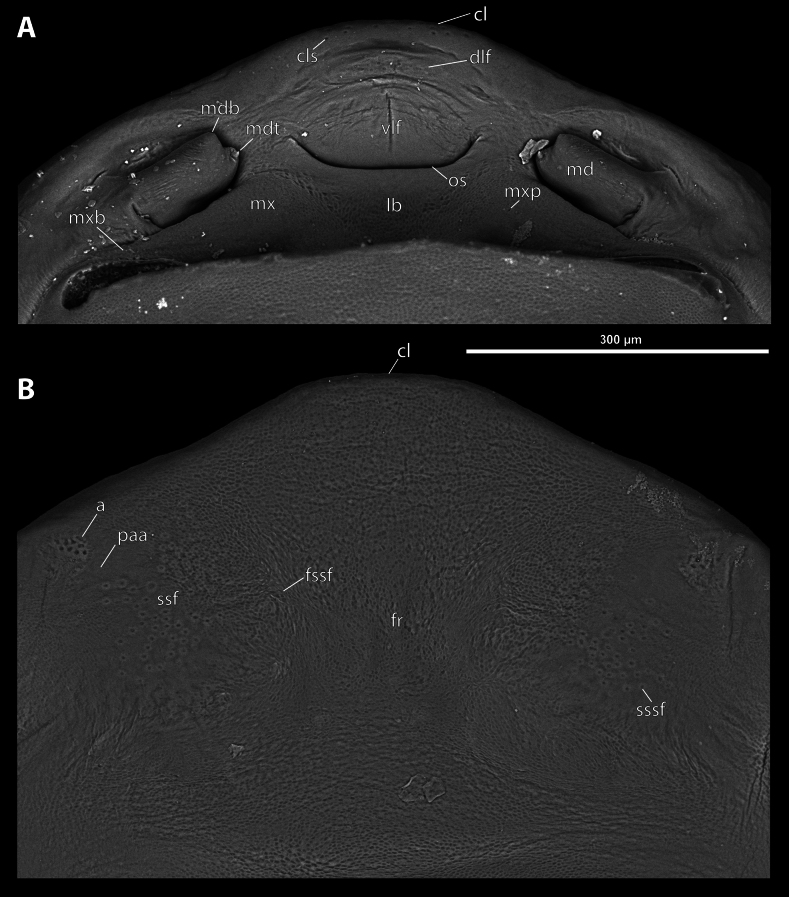
*Deltoxenos indonesiensis* Benda & Straka, sp. female cephalothorax, SEM micrographs. A. Detail of head capsule, ventral side; B. Detail of head capsule, dorsal side. Abbreviations: a – vestigial of antenna, cl – clypeal area, cls – clypeal sensillum, dlf – dorsal labral field of labral area, fr – frontal region, fssf – furrow of supra-antennal sensillary field, md – mandible, mdb – mandibular bulge, mdt – mandibular tooth, mx – vestige of maxilla, mxb – maxillary base, mxp – vestige of maxillary palp, lb – labial area, os – mouth opening, paa – periantennal area, ssf – supra-antennal sensillary field, sssf – sensillum of supra-antennal sensillary field, vlf – ventral labral field of labral area.

Figs 5, 6A–D, 7A–D, 8A, B

#### Type material.

***Holotype*** • ♀ (NMPC), Indonesia: Tanimbar Islands, Yamdena isl. 20km NE Soumiaki, 150m, 15.iv.2007, host: *Delta pyriforme miraculum* Gusenleitner, 2008, M. Obořil lgt., voucher code: PsIND1. ***Paratypes*** • ♀ (OLML), data the same as holotype, voucher code: PsMr2 • ♀ (OLML), data the same as holotype, host: *Delta pyriforme miraculum* Gusenleitner, 2008 (host specimen is paratype), voucher code: PsMr1 • 2♀ (OLML), Indonesia: Tanimbar Islands, Yamdena isl. 21 km N of Saumlaki, 150m, 11.xii.2005, host: *Delta pyriforme miraculum* Gusenleitner, 2008, S. Jákl lgt., voucher codes: PsMr3a, PsMr3b.

#### Diagnosis of female cephalothorax.

This species is diagnosed by a combination of features and differs from *D. reginus* in several characters. In *D. reginus*, the prosternum is differentiated, anteriorly pale brown with smooth or slightly wrinkled surface, posteriorly dark and reticulated, whereas in *D. indonesiensis* it is completely reticulated and dark. The ventral field of the labrum is slightly wider than long in *D. indonesiensis* (vlf, Fig. 8A) but distinctly wider than long in *D. reginus*, transversely elongated, elliptic (vlf, Fig. 15A). The mandible and maxilla of *D. indonesiensis* is dark brown and significantly darker than in *D. reginus*. The cuticle of the mandible is predominantly smooth in *D. indonesiensis* and laterally with longitudinal furrows but completely wrinkled in *D. reginus*. The periantennal area is narrow in *D. indonesiensis* but slightly expanded in *D. reginus* (paa, Fig. 15B).

The area of the mouthparts in front of the birth opening is shortened and the maxillae are reduced compared to conditions found in *D. hajeki* and *D. maceki*. It differs from *D. hajeki* in the following features: frontal region distinctly reticulated (fr, Fig. 8B), labium longer than wide (lb, Fig. 8A). Differs from *D. rueppelli* by rounded and protruding clypeal lobe. Maxilla not well-developed, slightly bulging, triangular, slightly separated from labial area (mx, Fig. 8A); and meso- and metathorax transverse, distinctly elongated as in *D. reginus*.

#### Description of female cephalothorax.

***Shape and colouration*.** Size of cephalothorax of holotype: length 2.25 mm, width 1.78 mm. Range of size of cephalothorax: length 2.17–2.5 mm, width 1.78–2.0 mm (Fig. 6A, C). Cephalothorax variable in size, but always elongated, distinctly longer than wide. Constriction at pro-mesothoracic segmental border indistinctly visible, meso-metathoracic segmental border conspicuously constricted laterally (sbpm, sbmm, Fig. 6C). Abdominal segment I not protruding laterally, corner below spiracles rounded. Anterior head margin rounded, slightly protruding from remaining head capsule. Thorax elongated, very slightly widening posteriorly. Cephalothorax with conspicuously contrasting pale and dark colour pattern, predominantly dark ventrally and pale dorsally.

***Head capsule*.** Ca 1/3 as long as entire cephalothorax including lateral cephalic extension. Colouration forming specific pattern with predominantly pale parts and dark brown mandibles, maxillae, and labium. Surface of lateral extensions at site of reduced compound eyes smooth, laterally with only poorly visible longitudinal grooves, mostly pale brown (lehc, Fig. 6C). Clypeal area well delimited from labral area medially, poorly laterally; arcuate, clypeal lobe protruding from head capsule. Surface of clypeal area completely smooth with > 20 slightly exposed sensilla mainly concentrated on clypeal lobe on ventral side (cls, Fig. 8A). Dorsal side of clypeal area not well visible, smooth, and lacking sensilla (cl, Fig. 8B). Border between clypeal and frontal region distinct, distinguishable by cuticular surface structure. Frontal region distinctly reticulated (fr, Fig. 8B). Segmental border between head and prothorax indicated by very indistinct mesal furrow on dorsal side (sbhp, Fig. 7B) and by dark transverse stripe laterally (sbhp, Fig. 6D). Head and prothorax distinctly separated by birth opening ventromedially (bo, Fig. 7A) and laterally by suture (sbhp, Fig. 7A).

***Supra*-*antennal sensillary field*.** Reticulated to completely smooth, with dispersed sensilla inserted in cavities (sssf, Fig. 8B). Very slightly delimited by furrow on medial side, almost unrecognisable (fssf, Fig. 8B), surface of supra-antennal sensillary field and frontal region with different sculpture.

***Antenna*.** Preserved as well-defined area, with numerous vestigial sensilla, rounded plates and inconspicuous cavities (a, Fig. 8B). Edges of antennal area not defined, antennal torulus completely reduced. Periantennal area not expanded, reduced, smooth (paa, Fig. 8B).

***Labrum*.** Ventral field wider than long, elliptic. Dorsal field very slightly arcuate, flat, not raised, laterally not narrower than medially, 5× wider than long in midline (vlf, dlf, Fig. 8A). Dorsal field with ~10 sensilla inserted in cavities (dlf, Fig. 8A).

***Mandible*.** Mandibles anteromedially directed at angle of 25–40° (40° in holotype), enclosed in mandibular capsule. Mandibular bulge distinctly raised, anteriorly directed in straight line, blunt, not elongated; sensilla of mandibular bulge poorly visible (mdb, Fig. 8A). Cuticle of mandible predominantly smooth, laterally with longitudinal furrows. Mandibular tooth not curved, pointed anterodorsally, armed with several spines (mdt, Fig. 8A).

***Maxilla*.** Indistinctly developed, slightly bulging, triangular, indistinctly separated from labial area (mx, Fig. 8A). Mostly dark, cuticle smooth or slightly reticulated. Apical maxillary region not projecting beyond mandibular apex. Basal part connected with labium, laterally very slightly overlapping with mandible (mxb, Fig. 8A). Vestige of palp present, located medially on ventral side of maxilla (mxp, Fig. 8A). Maxillary base (mxb) distinctly produced anterolaterally as submaxillary groove. Space between prothoracic extension and head not extended (sbhp, mxb, Fig. 7A).

***Labium*.** Labial area between maxillae rather indistinct, delimited anteriorly by mouth opening and posteriorly by birth opening (lb, Fig. 8A). Labial area flat, narrower than long. Cuticular surface smooth to very slightly reticulated.

***Mouth opening*.** Medially straight, laterally arcuate, sclerotised along margin (os, Fig. 8A).

***Thorax*.** Pro-mesothoracic and meso-metathoracic borders visible ventrally as slightly to distinctly imprinted mesal furrows (sbpm, sbmm, Figs 6A, 7A). On dorsal side separated by conspicuous dark mesal furrows, distinctly contrasting with mostly pale thoracic segments (sbpm, sbmm, Figs 6B, 7B). Border between metathorax and abdomen indicated by ventral ridge or indicated by change in colour and cuticular sculpture. Cuticle of thoracic segments on ventral side reticulated or smooth, with dark papillae concentrated medially on mesosternum and metasternum. Prosternum differentiated, mostly dark, anteriorly with inconspicuous field with dozens of sensilla (psf, Fig. 7A, C). Mesosternum mostly dark, without spots, with dark papillae medially. Metasternum mostly dark, lighter medially, with two mesal areas of dark papillae (mtstp, Fig. 6C). All thoracic segments dorsally mostly pale, but darker laterally. Meso- and metathorax transverse, distinctly elongated.

***Abdominal segment I and spiracles.*** Setae and cuticular spines present on lateral region of abdominal segment I posterior to spiracle (Fig. 7D). Spiracles on posterior ~1/3 of cephalothorax, very slightly elevated, with lateral or dorsolateral orientation. Cephalothoracic part of abdominal segment I below spiracles dark brown on both sides (asI, Fig. 6B).

#### Host.

*Delta pyriforme miraculum* Gusenleitner, 2008.

#### DNA barcode sequence (GenBank).

MK431200.1 (voucher code: PsInd1) (Benda et al. 2021).

#### Phylogenetic relationships.

Sister species to *Deltoxenos* sp. from Thailand, related to *Deltoxenos rueppelli* (Benda et al. 2021).

#### Distribution.

Indonesia.

#### Etymology.

The specific epithet refers to Indonesia, the geographic region where the species was collected.

### ﻿ Deltoxenos maceki

Taxon classificationAnimaliaStrepsipteraXenidae

Benda & Straka sp. nov.

0A586556-216F-5D3D-8AA7-C11720CB1953

https://zoobank.org/DCC5DD2B-F6DB-4F6A-83B2-788ECC9CBC3E

**Figure 9.**
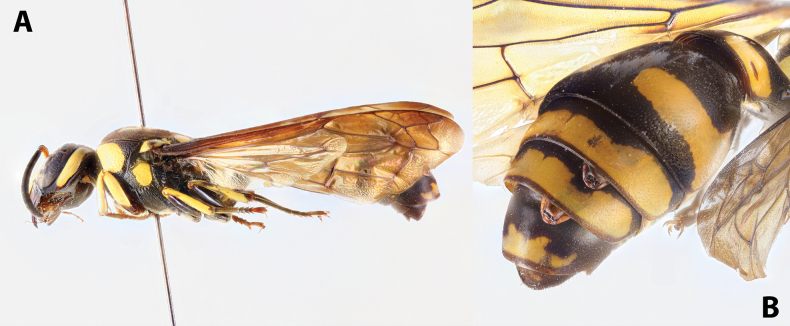
*Deltoxenos maceki* Benda & Straka, sp. nov., host, female cephalothorax. A. Female of *Pareumenes quadrispinosus* (Saussure, 1855), stylopised by *D. maceki* sp. nov., lateral view; B. Detail of host abdomen of *P. quadrispinosus*, with two female cephalothoraces inside.

**Figure 10.**
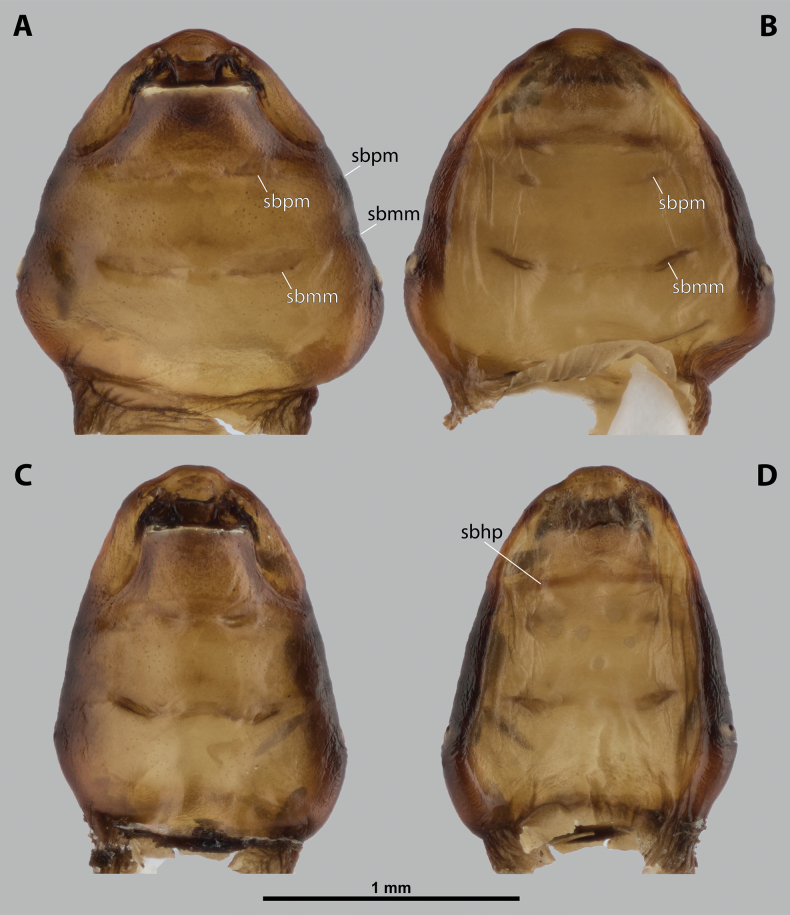
*Deltoxenos maceki* Benda & Straka, sp. nov., female, cephalothorax, photomicrographs. A. Paratype, voucher code: PsQd2b, ventral side of cephalothorax; B. Paratype, voucher code: PsQd2a, dorsal side of cephalothorax; C, D. Holotype, voucher code: PsPa1a, dorsal and ventral side of cephalothorax. Abbreviations: sbhp – segmental border between head and prothorax, sbmm – segmental border between mesothorax and metathorax, sbpm – segmental border between prothorax and mesothorax.

**Figure 11.**
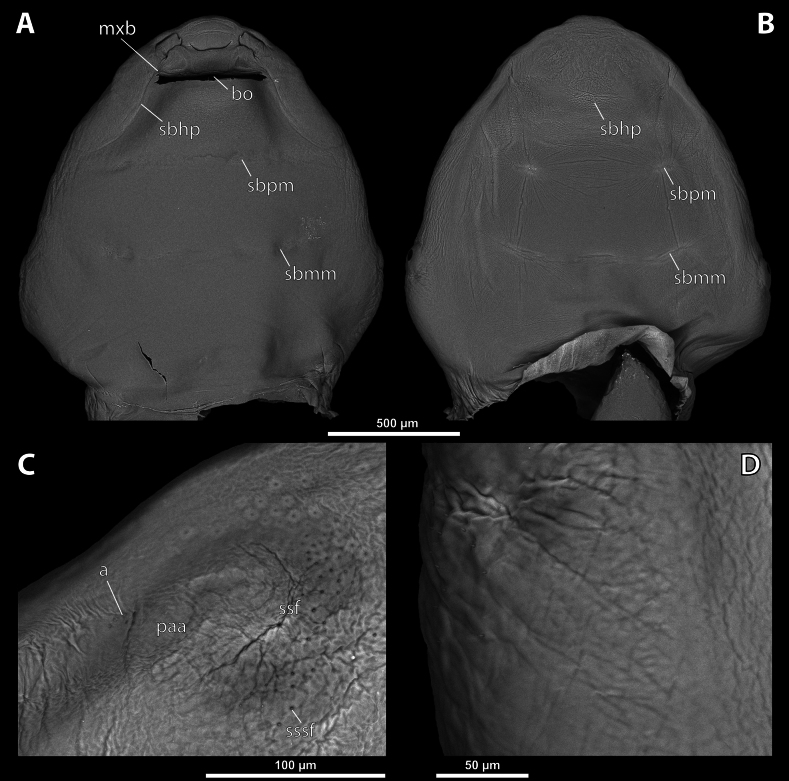
*Deltoxenos maceki* Benda & Straka, sp. nov., paratype, voucher code: PsQd2a, female cephalothorax, SEM micrographs. A. Ventral side; B. Dorsal side; C. Lateral part of head capsule, dorsal side; D. Posterolateral part of cephalothorax below spiracle, dorsal side. Abbreviations: a – vestigial of antenna, bo – birth opening, mxb – maxillary base, paa – periantennal area, sbhp – segmental border between head and prothorax, sbmm – segmental border between mesothorax and metathorax, sbpm – segmental border between prothorax and mesothorax, ssf – supra-antennal sensillary field, sssf – sensillum of supra-antennal sensillary field.

**Figure 12.**
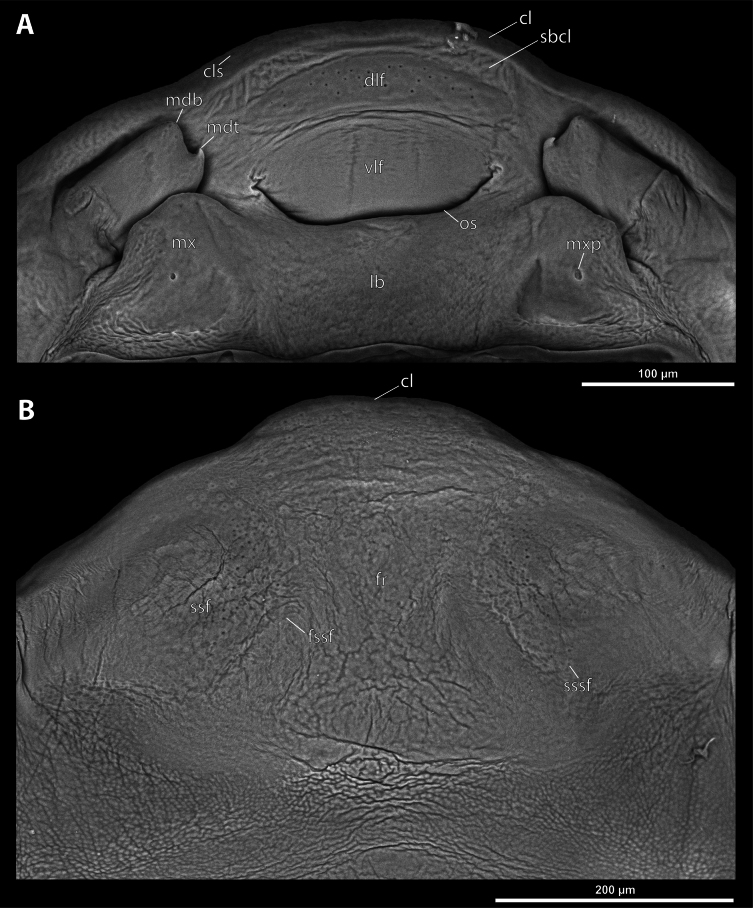
*Deltoxenos maceki* Benda & Straka, sp. nov., paratype, voucher code: PsQd2a, female cephalothorax, SEM micrographs. A. Detail of head capsule, ventral side; B. detail of head capsule, dorsal side. Abbreviations: cl – clypeal area, cls – clypeal sensillum, dlf – dorsal labral field of labral area, fr – frontal region, fssf – furrow of supra-antennal sensillary field, lb – labial area, mdb – mandibular bulge, mdt – mandibular tooth, mx – vestige of maxilla, mxp – vestige of maxillary palp, os – mouth opening, sbcl – segmental border between clypeus and labrum, ssf – supra-antennal sensillary field, sssf – sensillum of supra-antennal sensillary field, vlf – ventral labral field of labral area.

Figs 9A, B, 10A–D, 11A–D, 12A, B

#### Type material.

***Holotype*** • ♀ (NMPC), Vietnam: Hoa-binh env., 9.vi.1986, host: *Pareumenes quadrispinosus* (Saussure, 1855), J. Macek lgt., voucher code: PsPa1a. ***Paratypes*** • ♀ (NMPC), data the same as holotype, from the same host specimen as holotype, voucher code: PsPa1b • 2♀ (OLML), Laos: Khammouan prov., Nakai env., 550m, 15.vi.2001, host: *Pareumenes quadrispinosus* (Saussure, 1855), E. Jendek lgt., voucher codes: PsQd2a, PsQd2b.

#### Additional material.

2EMP (NMPC), data the same as holotype, from the same host specimen as holotype.

#### Diagnosis of female cephalothorax.

This species is diagnosed by a combination of characters. Thorax not elongated as in *D. indonesiensis* and *D. reginus*, distinctly widening posteriorly, almost triangular, similar to *D. bidentatus* (Fig. 10A), but narrow in some specimens (Fig. 10C). Differs from *D. rueppelli* by rounded and protruding clypeal lobe (cl, Fig. 12), differs from other species in very conspicuous and wide space at border between clypeal and labral areas (sbcl, Fig. 12A) and in shape of maxilla, which partly overlap with mandible (mx, Fig. 12A). From *D. hajeki* distinguished by anteriorly exposed clypeal sensilla (cls, Fig. 12A) which are mainly concentrated ventrally on the clypeal lobe in *D. hajeki*; antennal torulus completely absent in *D. maceki* while slightly reduced but still present in *D. hajeki*; prosternum anteriorly distinctly reticulated without any sensilla × prosternum differentiated, anteriorly with field of sensilla in *D. hajeki*.

#### Description of female cephalothorax.

***Shape and colouration*.** Size of holotype cephalothorax: length 1.5 mm, width 1.16 mm. Size of cephalothorax: length 1.44–1.5 mm, width 1.18–1.46 mm (Fig. 10). Cephalothorax only slightly varying in length but distinctly variable in width (compare Fig. 10A with Fig. 10C). Constriction at pro-mesothoracic segmental border indistinctly visible, meso-metathoracic segmental border conspicuously constricted laterally (sbpm, sbmm, Fig. 10A). Abdominal segment I not protruding laterally, corner below spiracles rounded. Anterior head margin rounded, slightly protruding from remaining head capsule. Thorax not elongated, distinctly widening posteriorly or narrow in few specimens. Cephalothorax with conspicuously contrasting pale and dark colour pattern but predominantly pale.

***Head capsule*.** Ca 1/3 as long as entire cephalothorax including lateral cephalic extension. Colouration forming specific pattern with predominantly light parts and dark brown mandible and labium. Surface of lateral extensions at site of reduced compound eyes smooth, laterally with slightly wrinkled cuticle and indistinct longitudinal grooves, dark-coloured. Clypeal area well delimited from labral area, arcuate, clypeal lobe slightly protruded from head capsule. Border between clypeal and labral area distinct and wide (sbcl, Fig. 12A). Clypeal surface smooth to slightly wrinkled with distinctly exposed sensilla mainly concentrated anteriorly on clypeal lobe (cls, Fig. 12A). Dorsal side of clypeal area wrinkled (cl, Fig. 12B). Border between clypeal and frontal region indistinct but still present. Frontal region distinctly reticulated and wrinkled (fr, Fig. 12B). Segmental border between head and prothorax indicated by very indistinct mesal furrow on dorsal side (sbhp, Fig. 11B) and by distinct dark transverse stripe visible on colour photograph (sbhp, Fig. 10D). Head and prothorax distinctly separated by birth opening ventromedially (bo, Fig. 11A) and laterally by suture (sbhp, Fig. 11A).

***Supra*-*antennal sensillary field*.** Completely reticulated and wrinkled, with dispersed sensilla inserted in cavities (sssf, Figs 11C, 12B). Very slightly delimited on medial side, almost unrecognisable (fssf, Fig. 12B), surface of supra-antennal sensillary field and frontal region with different sculpture.

***Antenna*.** Preserved as poorly defined area, with numerous vestigial sensilla and inconspicuous cavities (a, Fig. 11C). Antennal torulus completely absent, no border present as between antenna and periantennal area. Periantennal area reticulated, wrinkled (paa, Fig. 11C).

***Labrum*.** Ventral field wider than long, elliptic. Dorsal field very slightly arcuate, flat, not raised, laterally not narrower than medially, 5× wider than long in midline (vlf, dlf, Fig. 12A). Dorsal field with ~25 sensilla inserted in cavities, sensilla dispersed laterally (dlf, Fig. 12A).

***Mandible*.** Mandibles anteromedially directed at angle of 40–45° (45° in holotype), enclosed in mandibular capsule. Mandibular bulge distinctly raised, elongated, directed anteriorly, with several sensilla (mdb, Fig. 12A). Cuticle of mandible almost completely smooth, without lateral longitudinal furrows. Mandibular tooth slightly curved, pointed anteriorly, not conspicuously armed with spines (mdt, Fig. 12A).

***Maxilla*.** Well-developed and prominent, separated from labial area (mx, Fig. 12A). Mostly dark, medially pale; cuticle smooth medially, reticulated laterally. Apical maxillary region not projecting beyond mandibular apex. Basal part connected with labium, anterior part slightly overlapping with mandible (mx, Fig. 12A). Vestige of palp present, conspicuous, located medially on ventral side of maxilla (mxp, Fig. 12A). Maxillary base distinctly produced anterolaterally as submaxillary groove. Space between prothoracic extension and head not widened (sbhp, mxb, Fig. 11A).

***Labium*.** Labial area between maxillae distinct, delimited anteriorly by mouth opening and posteriorly by birth opening (lb, Fig. 12A). Flat, approximately as long as wide. Cuticular surface very slightly reticulated.

***Mouth opening*.** Very variable, laterally arcuate, medially sinuate to straight, area around mouth opening dark (os, Fig. 12A).

***Thorax*.** Pro-mesothoracic and meso-metathoracic borders visible ventrally as slightly imprinted mesal furrows (sbpm, sbmm, Figs 10A, 11A). On dorsal side separated by conspicuous dark mesal furrows, distinctly contrasting with pale thoracic segments (sbpm, sbmm, Figs 10B, 11B). Border between metathorax and abdomen indicated by ventral ridge on ventral side or indicated by change in colour and cuticular sculpture. Cuticle of thoracic segments on ventral side mostly pale except prothorax, with conspicuous pigmented papillae. Prosternum differentiated, anteriorly distinctly reticulated but without any sensilla; prosternum with typical colour pattern – dark anteromedially and laterally. Mesosternum and metasternum with two areas of dark papillae (mstp, mtstp, Fig. 10A). Cuticular surface of prosternal and mesosternal dark spots smooth, without any papillae or reticulation. All thoracic segments mostly pale dorsally and laterally. Meso- and metathorax transverse, rarely slightly elongated.

***Abdominal segment I and spiracles.*** Setae and cuticular spines present on lateral region of abdominal segment I posterior to spiracle (cus, Fig. 11D). Spiracles on posterior ~1/3 of cephalothorax very slightly elevated, with anterolateral orientation. Cephalothoracic part of abdominal segment I below spiracles orange to dark brown on both sides (asI, Fig. 10B).

#### Host.

*Pareumenes quadrispinosus* (Saussure, 1855).

#### Phylogenetic relationships.

Unknown.

#### Distribution.

Vietnam, Laos.

#### Etymology.

Named after Jan Macek (National Museum of the Czech Republic, Prague), a dear colleague and friend, as well as a world expert on Hymeno­ptera, who collected part of the type material.

### ﻿ Deltoxenos reginus

Taxon classificationAnimaliaStrepsipteraXenidae

Benda & Straka sp. nov.

4A371A04-710D-51E7-B7C7-287524AE2B4D

https://zoobank.org/8FAA0A73-F074-4B38-971E-64675A30FD30

**Figure 13.**
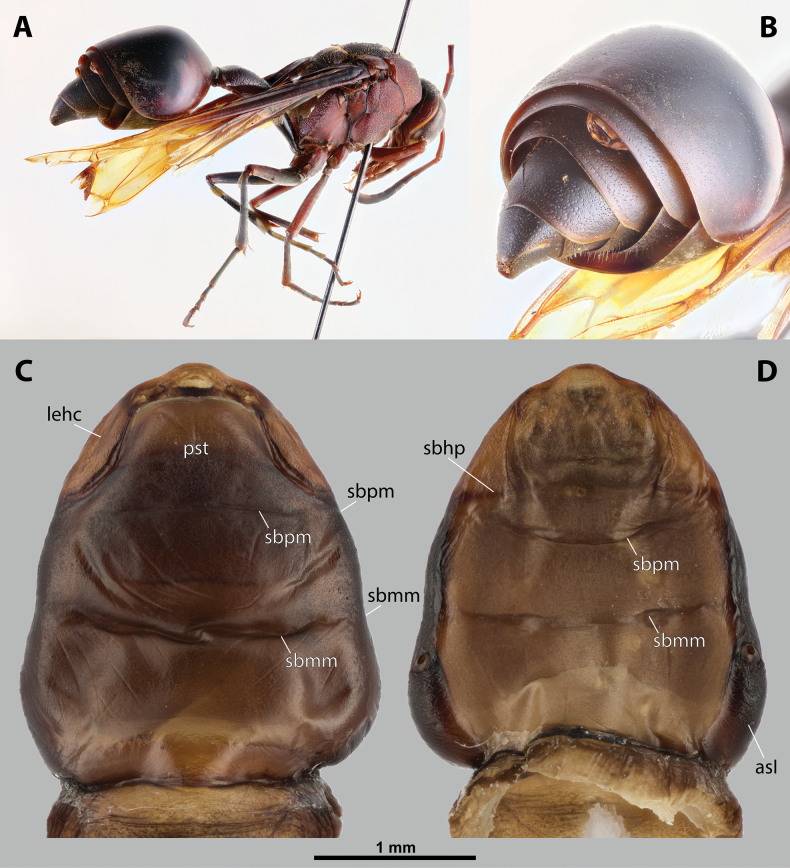
*Deltoxenos reginus* Benda & Straka, sp. nov., host, holotype, female cephalothorax, micrographs. A. Female of *Delta regina* (Saussure, 1852), stylopised by *D. reginus* sp. nov., lateral view; B. Detail of host abdomen of *D. regina*, with female cephalothorax inside; C. Holotype, ventral side of cephalothorax; D. Holotype, dorsal side of cephalothorax. Abbreviations: asI – abdominal segment I, lehc – lateral extension of head capsule, pst – prosternum, sbhp – segmental border between head and prothorax, sbmm – segmental border between mesothorax and metathorax, sbpm – segmental border between prothorax and mesothorax.

**Figure 14.**
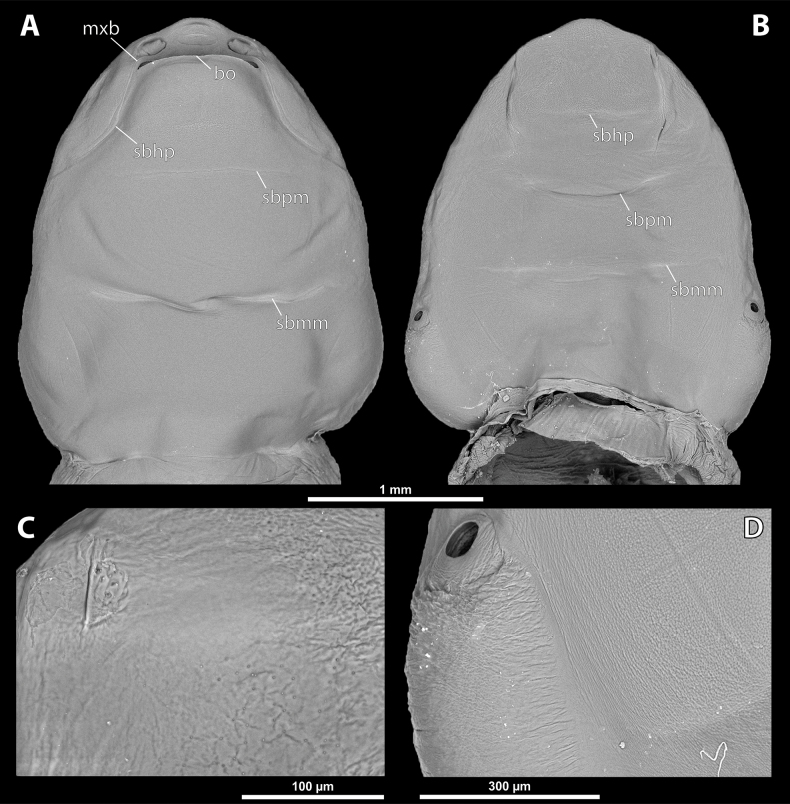
*Deltoxenos reginus* Benda & Straka, sp. nov., holotype, female cephalothorax, SEM micrographs. A. Ventral side; B. Dorsal side; C. Lateral part of head capsule, dorsal side; D. Posterolateral part of cephalothorax below spiracle, dorsal side. Abbreviations: bo – birth opening, mxb – maxillary base, sbhp – segmental border between head and prothorax, sbmm – segmental border between mesothorax and metathorax, sbpm – segmental border between prothorax and mesothorax.

**Figure 15.**
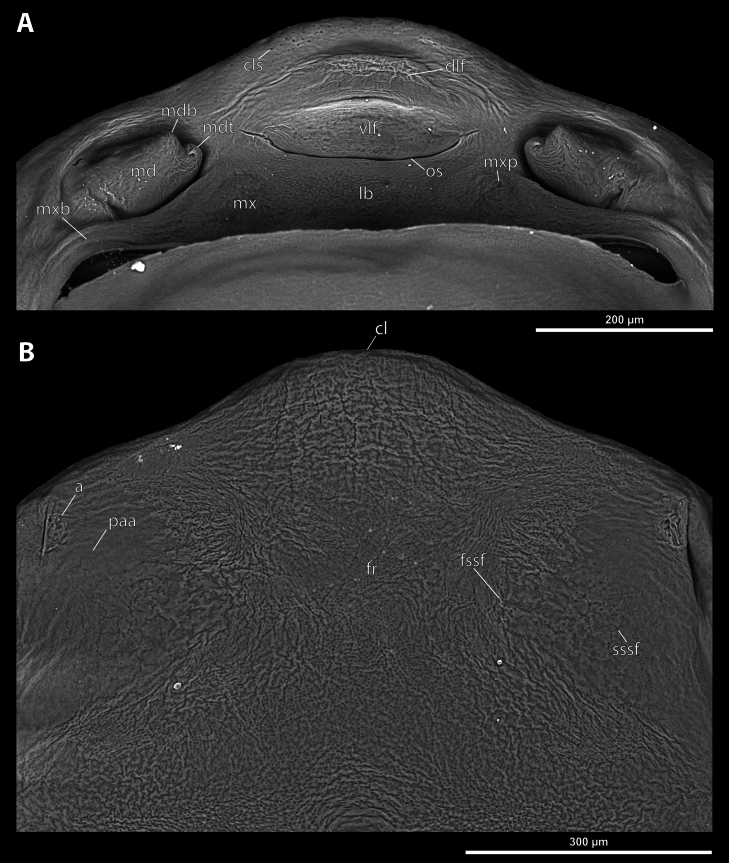
*Deltoxenos reginus* Benda & Straka, sp. nov., holotype, female cephalothorax, SEM micrographs. A. Detail of head capsule, ventral side; B. Detail of head capsule, dorsal side. Abbreviations: a – vestigial of antenna, cl – clypeal area, cls – clypeal sensillum, dlf – dorsal labral field of labral area, fr – frontal region, fssf – furrow of supra-antennal sensillary field, lb – labial area, md – mandible, mdb – mandibular bulge, mdt – mandibular tooth, mx – vestige of maxilla, mxb – maxillary base, mxp – vestige of maxillary palp, os – mouth opening, paa – periantennal area, vlf – ventral labral field of labral area, sssf – sensillum of supra-antennal sensillary field.

Figs 13A–D, 14A–D, 15A, B

#### Type material.

***Holotype*** • ♀ (KUNHM), Madagascar: Diego Suarez Prov., Mt. D´Ambre, Ambohitra Forest Preserve, 16.xi.1986, host: *Delta regina* (Saussure, 1852), J. Wenzel lgt.

#### Diagnosis of female cephalothorax.

For differentiation from *D. indonesiensis*, see diagnosis of the female cephalothorax of *D. indonesiensis* sp. nov. Area of mouthparts in front of birth opening shortened and maxillae reduced compared to *D. hajeki* and *D. maceki*. Differs from *D. rueppelli* by rounded and protruding clypeal lobe. Maxilla not well developed, slightly bulging, triangular, slightly separated from labial area (mx, Fig. 15A); meso- and metathorax distinctly elongated as in *D. indonesiensis*.

#### Description of female holotype cephalothorax.

***Shape and colouration*.** Size of cephalothorax: length 2.67 mm, width 2.27 mm (Fig. 13C). Cephalothorax large, elongated, distinctly longer than wide. Pro-mesothoracic segmental border constriction indistinctly visible, meso-metathoracic segmental border conspicuously constricted laterally (sbpm, sbmm, Fig. 13C). Abdominal segment I not protruding laterally, corner below spiracles rounded. Anterior head margin rounded, distinctly protruding from remaining head capsule. Thorax elongated, very slightly widening posteriorly. Cephalothorax with conspicuously contrasting light and dark colour pattern, predominantly dark ventrally and light dorsally.

***Head capsule*.** Length proportion of head/cephalothorax 0.34 including lateral cephalic extension. Colouration forming specific pattern with predominantly light parts and dark brown labium and slightly darker mandibles and maxillae. Surface of lateral extensions at site of reduced compound eyes smooth, laterally with only poorly visible longitudinal grooves; lateral extensions mostly light brown (lehc, Fig. 13C). Clypeal area well delimited from labral area medially, indistinctly laterally; clypeal lobe arcuate, distinctly protruding from head capsule. Surface of clypeal area smooth to slightly wrinkled, with > 25 exposed sensilla mainly concentrated on clypeal lobe on ventral side (cls, Fig. 15A). Dorsal side of clypeal area slightly wrinkled and lacking sensilla (cl, Fig. 15B). Border between clypeal and frontal region slightly visible, distinguishable by inconspicuous furrow. Frontal region distinctly reticulated and wrinkled (fr, Fig. 15B). Segmental border between head and prothorax indicated by very indistinct mesal furrow on dorsal side (sbhp, Fig. 14B) and by distinct dark transverse stripe (visible on colour photograph, sbhp, Fig. 13D). Head and prothorax distinctly separated by birth opening ventromedially (bo, Fig. 14A) and laterally by suture (sbhp, Fig. 14A).

***Supra*-*antennal sensillary field*.** Wrinkled to completely smooth, with dispersed sensilla inserted in indistinct cavities (sssf, Fig. 15B). Very slightly delimited by furrow on mesal side, indistinctly recognisable by cuticular surface structure (fssf, Fig. 15B), with supra-antennal sensillary field and frontal region with different sculpture.

***Antenna*.** Preserved as well-defined area, with several vestigial sensilla, rounded plates and inconspicuous cavities (a, Fig. 15B). Edges of antennal area poorly defined, antennal torulus completely reduced. Periantennal area slightly expanded, smooth (paa, Fig. 15B).

***Labrum*.** Ventral field distinctly wider than long, transversely elongated, elliptic. Dorsal field very slightly arcuate, flat, not raised, laterally not narrower than medially, 5× wider than long in midline (vlf, dlf, Fig. 15A). Sensilla on dorsal field very indistinctly visible.

***Mandible*.** Mandibles anteromedially directed at an angle of 30°, enclosed in mandibular capsule. Mandibular bulge distinctly raised, directed anteriorly, blunt, not elongated; sensilla of mandibular bulge poorly visible (mdb, Fig. 15A). Cuticle of mandible mostly wrinkled, laterally without longitudinal furrows. Mandibular tooth curved backwards, pointed posteriorly, armed with many small spines (mdt, Fig. 15A).

***Maxilla*.** Indistinct, slightly bulging, triangular, slightly separated from labial area (mx, Fig. 15A). Colouration dark to pale, with cuticle wrinkled, reticulated. Apical maxillary region not projecting beyond mandibular apex. Basal part connected with labium, laterally very slightly overlapping with mandible (mxb, Fig. 15A). Vestige of palp present, located medially on ventral side of maxilla, very indistinct (mxp, Fig. 15A). Maxillary base (mxb) distinctly produced anterolaterally as submaxillary groove. Space between prothoracic extension and head not extended (sbhp, mxb, Fig. 14A).

***Labium*.** Labial area between maxillae quite indistinct, delimited anteriorly by mouth opening and posteriorly by birth opening (lb, Fig. 15A). Flat, longer than wide. Cuticular surface wrinkled.

***Mouth opening*.** Very slightly arcuate, medially almost straight, sclerotised along margin (os, Fig. 15A).

***Thorax*.** Pro-mesothoracic and meso-metathoracic borders visible ventrally as slightly to distinctly imprinted mesal furrows (sbpm, sbmm, Figs 13C, 14A). On dorsal side separated by conspicuous dark mesal furrows, distinctly contrasted with mostly pale thoracic segments (sbpm, sbmm, Figs 13D, 14B). Border between metathorax and abdomen indicated by ventral ridge on ventral side or indicated by change in colour and cuticular sculpture. Cuticle of thoracic segments on ventral side predominantly reticulated, or smooth; dark pigmented papillae on mesosternum and metasternum almost absent. Pro­sternum differentiated, anteriorly light brown with smooth or slightly wrinkled surface, posteriorly dark and reticulated (pst, Fig. 13C); field of sensilla present. Mesosternum mostly dark, without spots. Metasternum predominantly dark, colour not distinctly lighter medially. All thoracic segments dorsally mostly pale, but darker laterally. Meso- and metathorax transverse, distinctly elongated.

***Abdominal segment I and spiracles.*** Setae and cuticular spines very rarely present on lateral region of abdominal segment I posterior to spiracle, cuticle mostly wrinkled (Fig. 14D). Spiracles on posterior ~1/3 of cephalothorax, very slightly elevated, with lateral or dorsolateral orientation. Cephalothoracic part of abdominal segment I below spiracles dark brown on both sides (asI, Fig. 13D).

#### Host.

*Delta regina* (Saussure, 1852).

#### Phylogenetic relationships.

Unknown.

#### Distribution.

Madagascar.

#### Etymology.

From the Latin substantive *regina*, meaning queen. The specific epithet *reginus* refers to the host species name. Adjective.

#### Comments.

Salt (1927) reported stylopised *Delta regina* (as Eumenes maxillosus (Degeer) var. reginus (Saussure)) from the same locality as the holotype (Fig. 17).

### ﻿ Deltoxenos rueppelli

Taxon classificationAnimaliaStrepsipteraXenidae

Kinzelbach, 1971

084B8738-B0C4-53E9-93C6-42C6314A8800

#### Material examined.

Kenya • 2♀ + EMP (OLML), Mwingi env., 7.iv.2007, host: *Delta emarginatum* (Linnaeus, 1758), M. Halada lgt., voucher code: PsDe8 • ♀ (OLML), E of Taveta, 7.iv.2007, host: *D. emarginatum*, M. Halada lgt., voucher code: PsDe9 • ♀ (OLML), SE Voi, 16.v.2007, host: *D. emarginatum*, M. Halada lgt., voucher code: PsDe21 • ♀ (OLML), Voi (Tsavo) env., 18.xi.1996, host: *Delta fenestrale* (Saussure, 1852), Mi. Halada lgt., voucher code: PsFe1 • 2EMP (OLML), Voi (Tsavo) env., 2.xii.1996, host: *D. fenestrale*, Mi. Halada lgt., voucher code: PsFe2 • ♀ + EMP (OLML), E of Mwingi, 14.05.2007, host: *D. fenestrale*, M. Halada lgt., voucher code: PsFe3 • ♀ (OLML), Taita Hills, Voi river, 14.iv.2007, *Delta hottentotum* (Saussure, 1852), M. Halada lgt., voucher code: PsHo2 • ♀ (OLML), data the same as voucher, voucher code: PsHo3 • ♀ (OLML), Voi (Tsavo) env., 18.xi.1996, *Delta l. lepeletierii* (Saussure, 1852), Mi. Halada lgt., voucher code: PsLp2 • ♀ (OLML), Taita Hills, Voi river, 14.iv.2007, *D. l. lepeletierii*, M. Halada lgt., voucher code: PsLp3; Kongo • ♀ (OLML), Bukama env., 1499 m, 15.viii.1923, *Delta lepeletierii formosum* (Saussure, 1852), collector not mentioned, voucher code: PsFo1; Malawi • ♀ (KUNHM), Blantyre, 4.iv.1967, host: *D. emarginatum*, Ch. Michener lgt., voucher code: PsDe6 • ♀ + 2MP (OLML), Selima env., 80 km E Lilongwe, 4.i.2002, *D. l. lepeletierii*, J. Halada lgt., voucher code: PsLp1 • ♀ (OLML), Nkhata Bay, Malawi Lake, 1.i.2001, host: *Delta tropicale* (Saussure, 1852), J. Halada lgt., voucher code: PsDe13; Namibia • 2♀ (NMPC), Karas prov., Seeheim 10km W, 717m, 05.iv.2017, host: *Delta caffrum* (Linnaeus, 1767), J. Halada lgt., voucher code: PsDe7 • 2EMP (NMPC), Khorixas 50 km NEE, Kunene prov., 1130 m, 25.iii.2014, *D. l. lepeletierii*, J. Halada lgt., voucher code: PsDe5; Republic of South Africa • EMP (OLML), North Cape, 27 km S of Strynenburg, 23.i.2000, host: *D. caffrum*, J. Halada lgt., voucher code: PsCa1 • 1EMP (OLML), Mpumalanga, 40 km SW Komatipoor, 2.i.2004, host: *D. emarginatum*, J. Halada lgt., voucher code: PsDe19 • ♀ (OLML), 35 km SE Makchado, Limpopo prov., 720 m, 14.xii.2009, *D. l. formosum*, J. Halada lgt., voucher code: PsFo2; Tanzania • 2♀ + MP (NMPC), Morogoro 50km SW, 450m, 12.i.2007, host: *D. caffrum*, J. Halada lgt., voucher code: PsDe2 • ♀ (NMPC), Rukwa, 30km NNW Mpanda, 1300m, 31.xii.2006, host: *D. emarginatum*, M. Kadlecová lgt., voucher code: PsDe1 • ♀ (NMPC), Gumbiro 30km N, Ruvuma pr., 980 m, 6.xii.2018, host: *Delta* sp., J. Halada lgt., voucher code: PsDe22; Yemen • 2♀ (NMPC), SE Bajil, Jabal Bura’A, 560 m, 22.iii.2017, *D. fenestrale*, D. Král lgt., voucher code: PsFe4; Zambia • MP (OLML) 150 km S Mwinilunga, 1100 m, 2.xi.2008, *D. hottentotum*, M. Halada lgt., voucher code: PsHo1 • ♀ (OLML), 100 km W Solwezi, 1400 m, 10.xi.2005, host: *D. hottentotum*, M. Halada lgt., voucher code: PsHo4 • ♀ (OLML), 150 km S Mwinilunga, 1100m, 2.xi.2008, *D. emarginatum*, M. Halada lgt., voucher code: PsDe17 • ♀ (OLML), data the same as voucher PsDe17, voucher code: PsDe18 • ♀ (OLML), 150 km S Mwinilunga, 1100 m, 2.xi.2008, host: *D. emarginatum*, M. Halada lgt., voucher code: PsDe10 • ♀ (OLML), 150 km S Mwinilunga, 1100 m, 18.x.2008, host: *D. emarginatum*, M. Halada lgt., voucher code: PsDe11 • ♀ (OLML), 100 km W Solwezi, 1400 m, 10.xi.2005, *Delta phthisicum* (Gerstäcker, 1857), M. Halada lgt., voucher code: PsDe12 • MP (OLML), 150 km S Mwinilunga, 1100 m, 2.xi.2008, *Delta pulcherrimum* Schulthess, 1910, M. Halada lgt., voucher code: PsPu1 • EMP (OLML), W of Solwezi, 1300 m, 22.x.2008, host: *D. tropicale*, Ma. Halada lgt., voucher code: PsDe14 • 2♀ + EMP (OLML), 10 km S Mazambuka, 28.xii.2002, host *D. tropicale*, J. Halada lgt., voucher code: PsDe15; Zimbabwe • MP (OLML), Chimanimani NP, 14.xii.1998, host: *D. emarginatum*, J. Halada lgt., voucher code: PsDe20.

#### Diagnosis of female cephalothorax.

**Figure 16.**
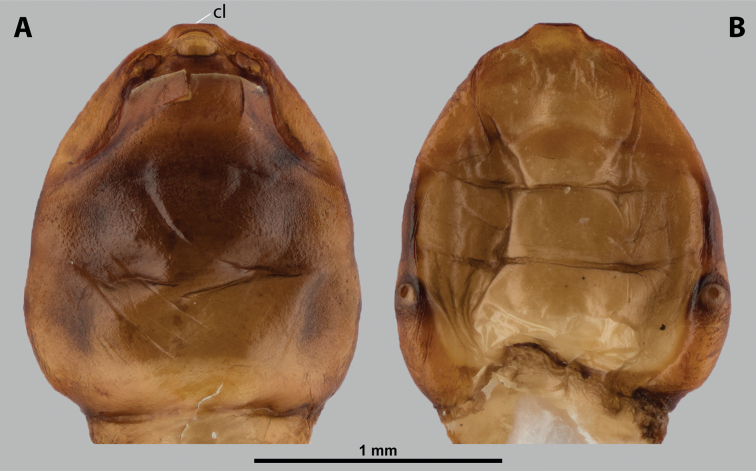
*Deltoxenos rueppelli* Kinzelbach, 1971, female, cephalothorax, photomicrographs. A. Ventral side of cephalothorax; B. Dorsal side of cephalothorax. Abbreviations: cl – clypeal area.

Size of cephalothorax very variable: length 1.48–2.83 mm, width 1.2–2.43 mm. Clypeal lobe distinctly blunt, truncate (cl, Fig. 16A).

**Figure 17.**
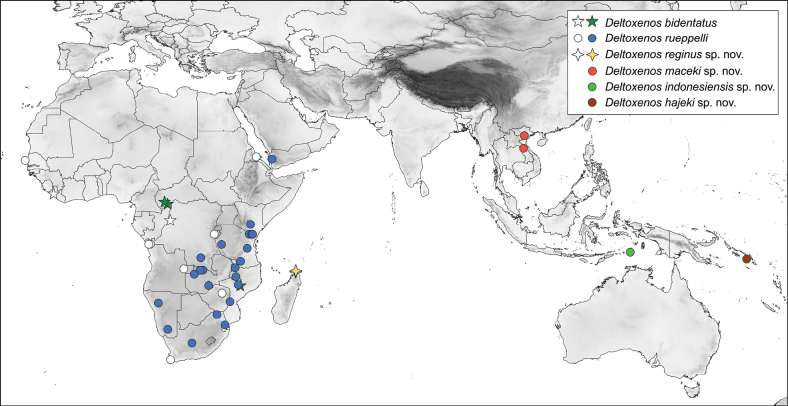
Distribution map of known *Deltoxenos* Benda, Pohl, Nakase, Beutel & Straka, 2022 species parasitizing wasps of the tribe Eumenini. White dots and crosses indicate data from published literature, while coloured symbols denote newly acquired distribution records.

#### Hosts.

*Delta emarginatum* (Linnaeus, 1758), *D. fenestrale* (Saussure, 1852) (type host) (Kinzelbach 1971a, Luna de Carvalho 1978); *D. caffrum* (Linnaeus, 1767) (Benda et al. 2021); *D. h. hottentotum* (Saussure, 1852), *D. lepeletierii formosum* (Saussure, 1852), *D. l. lepeletierii* (Saussure, 1852), *D. phthisicum* (Gerstäcker, 1857), *D. pulcherrimum* Schulthess, 1910, *D. tropicale* (Saussure, 1852) (this study).

#### DNA barcode sequences (GenBank).

MK431197.1 (voucher code: PsDe1), MN914584.1 (voucher code: PsDe2).

#### Phylogenetic relationships.

One of the species of *Deltoxenos* with the maximum of derived features, sister to *D. indonesiensis* + *Deltoxenos* sp. from Thailand (Benda et al. 2021).

#### Distribution.

Angola (Luna de Carvalho 1967); Democratic Republic of the Congo (Salt and Bequaert 1929, Luna de Carvalho 1978); Eritrea (type locality) (Kinzelbach 1971a); Kenya, Namibia, Yemen (Benda et al. 2022b); Republic of South Africa (Székessy 1959); Senegal (Hofeneder and Fulmek 1942); Tanzania (Benda et al. 2021); Zimbabwe (Salt 1927); Congo, Kenya, Malawi, Namibia, Zambia (this study).

#### Comments.

Benda et al. (2021) incorrectly lists distribution in Ethiopia although the type locality (Massawa) is located in Eritrea.

### ﻿Key to *Deltoxenos* species parasitising wasps of the tribe Eumenini (cephalothorax of female)

**Table:** 

1	Cephalothorax triangular, meso- and metathorax not elongated; apical maxillary region projecting beyond mandibular apex (Benda et al. 2022b: fig. 47C)	***Deltoxenos bidentatus* Pasteels.**
–	Cephalothorax more torpedo-shaped, meso- and metathorax elongated; apical maxillary region not projecting beyond mandibular apex (mx, Fig. 4A)	**2**
2	Clypeal lobe blunt, truncated (cl, Fig. 16A)	***Deltoxenos rueppelli* Kinzelbach**
–	Clypeal lobe rounded and protruding (cl, Figs 4A, 8A)	**3**
3	Maxilla well-developed and prominent, separated from labial area (mx, Figs 4A, 12A)	**4**
–	Maxilla not well-developed, slightly bulging, triangular, indistinctly separated from labial area (mx, Figs 8A, 15A)	**5**
4	Single dark spot placed posteriorly on prosternum (pds, Fig. 2A, C), double dark spot (mds, Fig. 2A, C) mesally on mesosternum, clypeal sensilla distinctly exposed and mainly concentrated ventrally on clypeal lobe (cls, Fig. 4A), antennal torulus reduced but still present (a, Fig. 4B)	***Deltoxenos hajeki* sp. nov.**
–	Dark spots absent (Fig. 10A, C), clypeal sensilla almost completely missing on ventral side (cls, Fig. 12A), antennal torulus completely absent (a, Fig. 11C)	***Deltoxenos maceki* sp. nov.**
5	Prosternum anteriorly light brown, with smooth or slightly wrinkled surface (pst, Fig. 13C), ventral field of labrum distinctly wider than long, transversely elongated, elliptic (vlf, Fig. 15A), cuticle of mandible completely wrinkled (md, Fig. 15A)	***Deltoxenos reginus* sp. nov.**
–	Prosternum posteriorly dark and reticulated (Fig. 6A, C), ventral field of labrum slightly wider than long (vlf, Fig. 8A), cuticle of mandible mostly smooth, laterally with longitudinal furrows (md, Fig. 8A)	***Deltoxenos indonesiensis* sp. nov.**

## ﻿Discussion

*Deltoxenos* is a relatively small genus comprising eight species, with a distribution spanning the Old World and Australasian region (Benda et al. 2024). Phylogenetically, *Deltoxenos* is deeply nested within Xenidae and is of Afrotropical origin, with *Xenos* Rossi identified as its sister group (Benda et al. 2019). Although a recent dispersal event from Afrotropics to the Palearctic, Indomalayan, and Australasian occurred in the evolutionary history of this lineage, most described species are still known primarily from Africa and Japan (Benda et al. 2022b). This dispersal likely followed a host switch from the wasp tribe Odynerini to Eumenini, which may have opened a novel ecological niche, facilitating diversification and range expansion (Benda et al. 2019). Moreover, a recently described *Deltoxenos* species from wasps of the genus *Zethus* Fabricius in Africa suggests that evolution of host specificity within the genus may be even more complex than we expected (Benda et al. 2024).

A parallel dispersal pattern is observed in the genus *Paraxenos* Saunders, which during the past few million years switched to digger wasp species of *Bembecinus* Costa as hosts, enabling its spread in the same direction as in *Deltoxenos* from the Afrotropical-Palearctic through the Indomalayan region all the way to Australia. These patterns support the hypothesis that the Afrotropics served as a key source of xenid diversity for the Old World and Australia from the Miocene to the present (Benda et al. 2019).

In addition to the description of four new Xenidae species, we also provide host records for species with hitherto unknown parasitism by strepsipteran species. These include two genera (or species), *Phimenes solomonis* and *Pareumenes quadrispinosus*, and five species of the genus *Delta*, including two subspecies: *D. hottentotum*, *D. lepeletierii formosum*, *D. lepeletierii lepeletierii*, *D. phthisicum*, *D. pulcherrimum*, and *D. tropicale*. These discoveries suggest, in context with our previous work and with data from older publications, that wasp species of Eumenini are more frequently parasitised than expected and more vigilance is needed when handling material of species of Vespidae (Salt 1927; Salt and Bequaert 1929; Benda et al. 2021). This applies to many genera of the Eumeninae, especially those that are highly diversified. For example, the genus *Delta* comprises approximately 50 species distributed throughout the Old World and Australasian regions, with one species adventive in North America and the Antilles (Carpenter 2008). Of these, 21 species are known from the Oriental region (Nguyen 2015), where there is a high probability of occurrence of additional, yet undiscovered species of *Deltoxenos*. However, the taxonomy of *Delta* wasps remains in a confused state, primarily due to the presence of numerous subspecies (Carpenter 2008). According to Benda et al. (2022b), *Deltoxenos* represents the genus within Xenidae with the greatest potential for the discovery of undescribed species. In particular, host genera with high species diversity may indicate a corresponding hidden diversity of undescribed *Deltoxenos* species. This is the case, for example, with the genus *Mischocyttarus* Saussure (host of *Xenos*), which comprises approximately 250 described species, and with *Bembix* Fabricius (host of *Paraxenos*) with ~300 species. Despite this host diversity, only a few associated strepsipteran species have been described to date, suggesting that many more probably remain to be discovered (Benda et al. 2022a, 2023).

However, some host-parasite interactions remain enigmatic and unresolved. Esaki (1931) described *Pseudoxenos iwatai* from *Eumenes japonica* Saussure, recently synonymised with *Oreumenes decoratus* (Smith, 1852) by Yamane (1990). Although, Benda et al. (2022b) preliminary assigned *P. esakii* to the genus *Deltoxenos* based on host specificity, Maeta (1971) reported five host genera for this species (*Eumenes*, *Ancistrocerus*, *Odynerus*, *Rygchium*, and *Anterhynchium*). However, the xenid specimen parasitising the latter host (*Ante­rhynchium flavomarginatum* Smith), belongs – based on a molecular phylogeny of Xenidae – to an entirely distinctive lineage, currently recognised as *Macroxenos* (Benda et al. 2021, 2022b). Unfortunately, only a poor description of a male is known from the original host (*O. decoratus*) (Esaki 1931). Additional DNA sequences from individuals parasitising other hosts of *P. iwatai*, with a detailed morphological examination of female cephalothoraxes, are essential to clarify the systematic position of this species.

When describing new species, it is desirable to examine as many individuals as possible due to the variability of certain morphological characters – an aspect particularly critical for morphologically variable endoparasitic species (Nadler and León 2011). Proper documentation of such variability is also important, as traits like the shape of the cephalothorax can exhibit significant intraspecific variation, as clearly demonstrated in Fig. 10. For the delimitation of *Deltoxenos* species, key diagnostic characters include the colouration of the cephalothorax, the shape of the clypeal lobe, the shape of maxilla, the position of the clypeal sensilla, and the microsculpture of the cuticular surface, characters which are relevant in the context of diagnostic criteria outlined by Benda et al. (2022a). Interestingly, within the *Deltoxenos* species group parasitising Eumenini, *D. bidentatus* differs by its triangular cephalothorax shape. This is consistent with phylogenetic evidence, as molecular phylogenetics place it as the earliest-diverging species within this clade (Benda et al. 2021). Morphologically, *D. bidentatus* more closely resembles *Deltoxenos* species parasitising wasps of the tribe Odynerini, whereas species associated with Eumenini are typically torpedo-shaped, with elongated meso- and metathoraces. The distinct elongation is especially pronounced in species such as *D. indonesiensis* and *D. reginus*.

## Supplementary Material

XML Treatment for Deltoxenos bidentatus

XML Treatment for Deltoxenos hajeki

XML Treatment for Deltoxenos indonesiensis

XML Treatment for Deltoxenos maceki

XML Treatment for Deltoxenos reginus

XML Treatment for Deltoxenos rueppelli

## ﻿Additional information

### Use of AI

No use of AI was reported.

### Author ORCIDs

Daniel Benda https://orcid.org/0000-0002-5729-0411

Hans Pohl https://orcid.org/0000-0002-7090-6612

Rolf Beutel https://orcid.org/0000-0002-0433-7626

Jakub Straka https://orcid.org/0000-0002-8987-1245

### Data availability

All of the data that support the findings of this study are available in the main text.
